# Single-Cell RNA Sequencing of Human Corpus Cavernosum Reveals Cellular Heterogeneity Landscapes in Erectile Dysfunction

**DOI:** 10.3389/fendo.2022.874915

**Published:** 2022-04-20

**Authors:** Dong Fang, Xiao-Hui Tan, Wen-Peng Song, Yang-Yang Gu, Jian-Cheng Pan, Xiao-Qing Yang, Wei-Dong Song, Yi-Ming Yuan, Jing Peng, Zhi-Chao Zhang, Zhong-Cheng Xin, Xue-Song Li, Rui-Li Guan

**Affiliations:** ^1^ Department of Urology, Peking University First Hospital, Beijing, China; ^2^ Institute of Urology, Peking University, Beijing, China; ^3^ Beijing Key Laboratory of Urogenital Diseases (male) Molecular Diagnosis and Treatment Center, Beijing, China; ^4^ Department of Dental Implant Center, Beijing Stomatological Hospital, School of Stomatology, Capital Medical University, Beijing, China; ^5^ Department of Radiation Medicine, Institute of Systems Biomedicine, School of Basic Medical Sciences, Peking University Health Science Center, Beijing, China; ^6^ Male Reproductive and Sexual Medicine, Department of Urology, The Second Hospital of Tianjin Medical University, Tianjin, China; ^7^ Institute of Urology, Tianjin Medical University, Tianjin, China

**Keywords:** endothelial cells, erectile dysfunction, fibroblasts, RNA-seq, smooth muscle cells, single-cell analysis

## Abstract

**Purpose:**

To assess the diverse cell populations of human corpus cavernosum in patients with severe erectile dysfunction (ED) at the single-cell level.

**Methods:**

Penile tissues collected from three patients were subjected to single-cell RNA sequencing using the BD Rhapsody™ platform. Common bioinformatics tools were used to analyze cellular heterogeneity and gene expression profiles from generated raw data, including the packages Seurat, Monocle, and CellPhoneDB.

**Results:**

Disease-related heterogeneity of cell types was determined in the cavernous tissue such as endothelial cells (ECs), smooth muscle cells, fibroblasts, and immune cells. Reclustering analysis of ECs identified an arteriole ECs subcluster and another one with gene signatures of fibroblasts. The proportion of fibroblasts was higher than the other cell populations and had the most significant cellular heterogeneity, in which a distinct subcluster co-expressed endothelial markers. The transition trajectory of differentiation from smooth muscle cells into fibroblasts was depicted using the pseudotime analysis, suggesting that the expansion of corpus cavernosum is possibly compromised as a result of fibrosis. Cell-cell communications among ECs, smooth muscle cells, fibroblasts, and macrophages were robust, which indicated that inflammation may also have a crucial role in the development of ED.

**Conclusions:**

Our study has demonstrated a comprehensive single-cell atlas of cellular components in human corpus cavernosum of ED, providing in-depth insights into the pathogenesis. Future research is warranted to explore disease-specific alterations for individualized treatment of ED.

## Introduction

Erectile dysfunction (ED) is defined as the inability to attain and/or maintain penile erection sufficient for satisfactory sexual performance, which exerts substantial effects on a certain proportion of men at least occasionally (1). This disease occurs in 3-76.5% of the global male population with an average prevalence of 30% and was found to be associated with aging, health status, and emotional function (2–4). Moreover, ED might be a warning sign of cardiovascular disease owing to their shared pathophysiological links and risk factors such as diabetes mellitus, endothelial dysfunction, and inflammation (5). The etiology is multifactorial and can be broadly classified into organic and psychogenic. Of the organic etiologies, vasculogenic (affecting blood supply), neurogenic (affecting innervation and nervous function), and endocrinologic (relating to endocrine factors) are common causes of ED. The mechanisms involved in the development of ED have been well investigated in the past decades, in which nitric oxide (NO) and soluble guanylate cyclase (sGC) in the intracellular cyclic guanosine monophosphate (cGMP) signaling are principally responsible (6). Although several theories have been proposed in the development of ED, few studies have assessed the cellular composition, intracellular communications, and molecular characteristics of human corpus cavernosum at the single-cell level.

High-throughput single-cell RNA sequencing (scRNA-seq) has been a frequently used tool to profile transcriptome information at the level of individual cells, which can characterize cellular heterogeneity and identify closely related cell populations (7). Recent advances in biotechnology and computational science have transformed the data analysis of the genome and transcriptome, holding vast potential in enhancing our understanding of cell and disease biology (8). For instance, many new approaches have been designed to facilitate a complete and detailed gene expression profile, such as identification of novel cell types and associated markers, prediction of developmental trajectories, and establishment of cell-cell interaction (9–11). Therefore, scRNA-seq can enable the transcriptomic profiling of thousands of cells in a single experiment and may uncover related pathological processes in a wide variety of tissues and organisms. The penile erectile tissue plays a key role in the erectile process, especially the smooth muscles and endothelium. Accordingly, a comprehensive transcriptomic analysis of cell types in ED would provide more in-depth information on its nature.

In the present study, we aimed to profile the transcriptome of single cells from patients with severe ED and generate a single-cell atlas of human corpus cavernosum. Furthermore, we compared the cell populations with a special focus on the subsets of smooth muscle cells (SMCs), fibroblasts, and endothelial cells (ECs).

## Methods

### Patient and Samples

The study protocols and tissue acquisition procedures were approved by the local institutional review board. Informed consent to participate in the study was provided by all subjects. Human cavernous tissue samples were collected from three adult patients with severe ED who received penile implants at our medical center. Fresh tissue samples were surgically removed and immediately dissected into fractions that were dissociated into single cells as described below.

### Sample Preparation for scRNA-Seq

Immediately after surgical removal, every tissue sample for scRNA-seq was washed with phosphate-buffered saline (PBS), cut into pieces about 1 mm^3^ on ice, and enzymatically digested at 37°C, with gentle rotation to obtain a single-cell suspension. Subsequently, DMEM complete medium was added for the termination of digestion, and then 70-μm and 40-μm cell strainers were used in sequence to filter the suspension. After the lysis of erythrocytes, the cells were centrifuged at 500 xg for 15 min and washed twice with PBS. Finally, the pellets were resuspended in ice-cold PBS with 0.05% bovine serum albumin, evaluated for cellular concentration and viability *via* BD Rhapsody™ Scanner (BD Biosciences, La Jolla, CA, USA), and processed for scRNA-seq using the BD Rhapsody™ platform.

### Single-Cell Transcriptome Capture, Library Construction, and Sequencing

All libraries for scRNA-seq were generated as described previously (12). Briefly, cell capture was achieved by random distribution across the microwells. Beads with oligonucleotide barcodes were prepared and loaded onto the cartridge, allowing a single bead to pair with a single cell. Cell-lysis buffer was used and then RNA molecules could hybridize to the beads. Beads were pooled together into a single tube to synthesize complementary DNA for reverse transcription. Each complementary DNA molecule was then labeled on the 5’ end with its unique molecular identifiers (UMI) and cell label information. Next, second-strand complementary DNA was synthesized and ligated with the adaptor for universal amplification. Random priming polymerase chain reaction was performed to enrich the 3’ end of the transcripts. The sequencing library for each sample was sequenced on the NovaSeq platform (Illumina, San Diego, CA, USA) with a 150-bp paired-end run.

### Raw Data Analysis

Raw sequencing data were processed and examined through the BD Rhapsody Whole Transcriptome Analysis pipeline. For clustering analysis and visualization, the gene expression matrices were analyzed in the R environment using the package Seurat (version 3.2.2) (13, 14). Quality-filtered reads were investigated to detect the sequences of cell labels and UMI, which were later mapped to the Genome Reference Consortium Human Build 38 for annotation. The dimensionality of filtered data was reduced *via* principal component analysis with the highly variable genes on the scaled data. The top 50 principal components were used for uniform manifold approximation and projection (UMAP) to visualize data in two dimensions. Clustering analysis was performed with the FindClusters function in the Seurat package. Differentially expressed genes (DEGs) for clusters or subtypes were identified using the FindAllMarkers function with default parameters. Major cell type was annotated with selected marker gene listed: ECs (*VWF*, *PODXL, EMCN*, *PECAM1*), SMCs (*ACTA2*, *TAGLN*, *MYH11*), fibroblasts (*DCN*, *LUM*, *COL1A2*, *PDGFRA*, *IGF1*), T cells (*CD2*, *CD3D/E/G*), neutrophils (*S100A8/9*), macrophages (*CD163*, *C1QA*, *C1QB*, *C1QC*), monocytes (*CD14*, *FCGR3A*), natural killer cells (*NKG7*, *GZMA*), neural cells (*SOX10*, *PLP1*), mast cells (*TPSB2*, *TPSAB1*), B cells (*CD79A*, *IGHM*, *MZB1*).

### Pseudotime Trajectory Analysis

Pseudotime trajectories were constructed with the R package Monocle 2, which could determine the transcriptional dynamics among cell types and clusters (15). The cell trajectory and position with tree structure were plotted, in which the data were reduced to two dimensions using the discriminative dimensionality reduction with trees method. Significant genes of clusters along the pseudotime values were identified with the differential_Gene_Test function and visualized using the plot_pseudotime_heatmap function with the default parameters.

### Cell-Cell Communication Prediction Analysis

Cell-cell communication network was predicted *via* the computational tool CellPhoneDB (version 2.0), which served as a publicly available repository of potential receptor-ligand interaction (16). Cell type-specific interactions between ligands and receptors were analyzed and only those expressed in at least 10% of the cells for each clusters were included. The cluster labels of every cell were randomly permuted 1000 times to determine the expression levels of the interacting clusters.

### Cell Cycle Analysis

To score the cell cycle phases of every single cell, the Cell_Cycle_Scoring function in Seurat was used based on the expression of canonical marker genes (17). A total of 42 S phase genes and 54 G2/M phase genes were included in the analysis. Cell cycle phases (G1, S, and G2/M) were then assigned to every single cell.

### Statistical Analysis

Statistical analyses were performed using R software, version 4.1.2 (http://www.rproject.org). Gene Ontology (GO) enrichment and Kyoto Encyclopedia of Genes and Genomes (KEGG) pathway analyses were performed using the database for annotation, visualization, and integrated discovery (DAVID, version 6.8) to identify biological and functional processes associated with DEGs in each cluster. The P-value for each gene set was calculated by Fisher’s exact test and those less than 0.05 were considered statistically significant.

## Results

### Cell Populations in the Human Corpus Cavernosum

**Figure 1:**
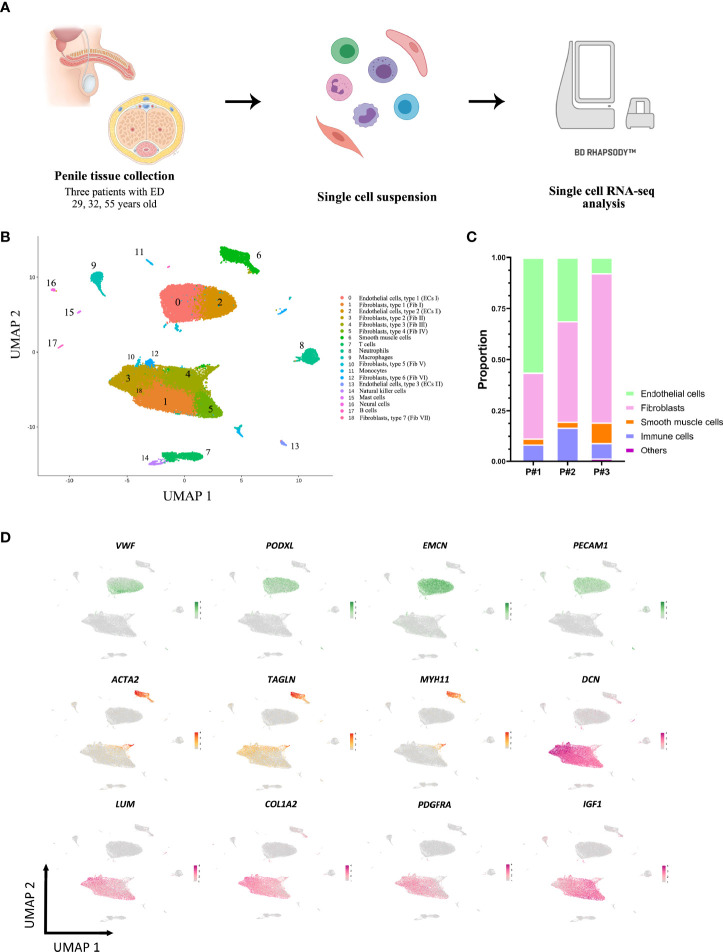
Overview of the single-cell landscape for corpus cavernosum in erectile dysfunction. **(A)** Schematic graph describing the workflow of the experiment. Human corpus cavernosum samples from three patients with erectile dysfunction were collected for single-cell RNA-seq. **(B)** A UMAP view and clustering analysis of combined single-cell transcriptome data from human corpus cavernosum (n = 37892). Clusters are distinguished by different colors with the general identity of each cell cluster shown on the right. **(C)** The cellular composition distribution for each patient sample. **(D)** Feature plots of expression distribution for selected genes. Expression levels for each cell are color-coded and overlaid onto the UMAP plot. Cell types were mainly classified as endothelial cells (green), smooth muscle cells (orange), and fibroblasts (pink). UMAP, uniform manifold approximation and projection.

To investigate cellular heterogeneity in ED at single-cell resolution, we collected 3 tissue samples from 3 distinct patients **(** **Table 1** **)** and performed scRNA-seq. After standard data processing and quality control procedures, we obtained transcriptomic profiles for 37,892 cells **(** **Figure 1A** **)**. Principal cell clusters were classified using an unsupervised graph-based clustering strategy, acquiring a comprehensive UMAP plot of the cellular composition **(** **Figures 1B**; **S1**, **S2** **)**. Cells with similar profiles were annotated based on the expression of lineage-specific marker genes, which consisted of ECs (C0, C2, C13), fibroblasts (C1, C3, C4, C5, C10, C12, C18), SMCs (C6), T cells (C7), neutrophils (C8), macrophages (C9), monocytes (C11), natural killer cells (C14), mast cells (C15), neural cells (C16), and B cells (C17). The proportions of these cell lineages in the human cavernous tissue from three patients were evaluated, in which ECs, SMCs, fibroblasts accounted for 31.7%, 5.3%, 51.5% on average **(** **Figure 1C** **)**. The lineage-specific marker genes for the majority of sequenced cells were as follows: ECs (*VWF, PODXL, EMCN, PECAM1*), fibroblasts (*DCN, LUM, COL1A2, PDGFRA, IGF1*), and SMCs (*ACTA2, TAGLN, MYH11*) **(** **Figure 1D** and **Table S1** **)**. ECs and fibroblasts were categorized into three clusters and seven clusters, the diversity of which was mainly attributed to their cellular heterogeneity. The information of DEGs in each cluster was summarized in **Table S2**.

**Table 1:** Characteristics of patients included in this study.

Patient	Diagnosis	Age (years)	BMI (kg/m²)	Possible mechanism	Disease severity*
Patient #1	Erectile dysfunction	29	29.4	Vasculogenic (primary disease)	Severe
Patient #2	Erectile dysfunction	55	30.4	Neurogenic (surgical injury)	Severe
Patient #3	Erectile dysfunction	32	23.2	Vasculogenic (pelvic trauma)	Severe

*The disease severity of erectile dysfunction was classified by international index of erectile function.

BMI, body mass index.

### Molecular Signatures of Endothelial Subpopulations

**Figure 2:**
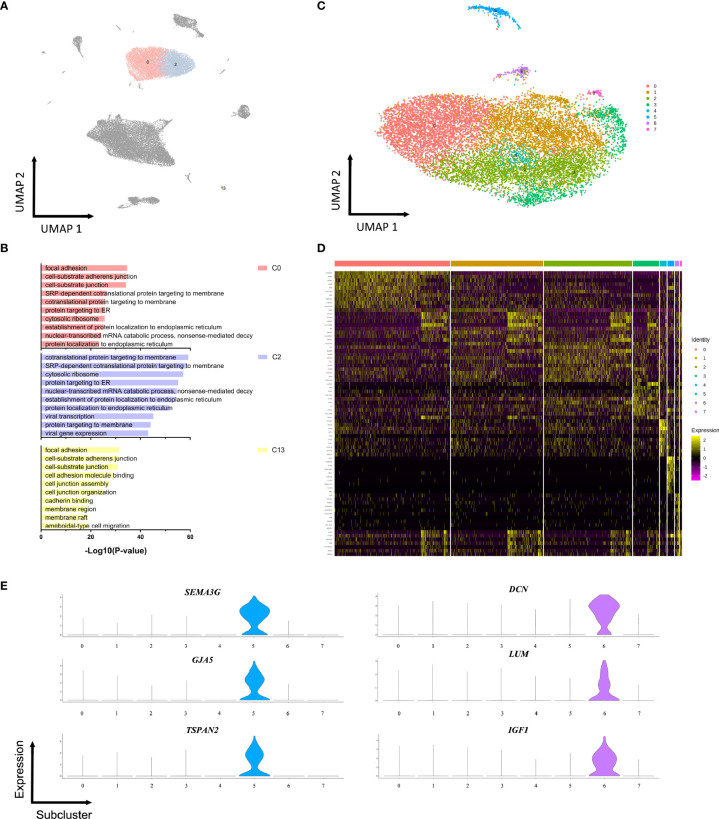
Endothelial subpopulations display specific functional transcriptomic signatures. **(A)** 13101 endothelial cells (clusters 0, 2, 13) were highlighted and colored in the UMAP plot of all clusters. **(B)** Functional enrichment analysis with GO terms was performed with the significantly up-regulated genes in three endothelial subpopulations. **(C)** Endothelial cells were extracted and reclustered into 7 subclusters plotted in a UMAP map. **(D)** Heatmap depicting differentially expressed genes among endothelial subclusters. **(E)** Expressions of *SEMA3G*, *GJA5*, *TSPAN2*, *DCN*, *LUM*, and *IGF1* in each subcluster.

As illustrated in **Figure 2A**, three distinct endothelial cell populations were identified, termed endothelial cell, type 1-3 (ECs I-III). Pan-endothelial markers (*PECAM1*, *CDH5*, *TIE1*) were expressed in all populations of ECs. A number of factors that are indispensable for arterial differentiation showed an abundant expression level in ECs III (e.g. *SOX17*, *HEY1*, *SEMA3G*). GO enrichment analysis of the DEGs identified cell type-specific processes **(** **Figure 2B** **)**. For instance, ECs III (C13) participated in focal adhesion and cell junction while ECs II (C2) were more likely to be involved in protein targeting and protein localization. To determine how the molecular signatures of ECs were altered in patients with ED, we performed unbiased clustering on endothelial subpopulations and observed further heterogeneity with 7 subclusters **(** **Figure 2C** and **Table S3** **)**. Two distinct cell subclusters (sC5, sC6) were identified and exhibited unique gene expression patterns compared with the others **(** **Figure 2D** **)**. In particular, the expression levels of arteriole markers (*HEY1*, *SERPINE2*, *SEMA3G*, *GJA5*) were exclusively enriched in the endothelial sC5 and similar to the transcriptome profile of ECs III (C13). Interestingly, our results showed a strong enrichment of canonical fibroblast-specific markers (*DCN*, *LUM*, *IGF1*) in the endothelial sC6 **(** **Figure 2E** **)**. Conversely, the other five subclusters expressed partially overlapping markers, suggesting a continuous phenotype gradient rather than the presence of true endothelial subpopulations.

### Heterogeneity of Fibroblasts and SMCs

**Figure 3:**
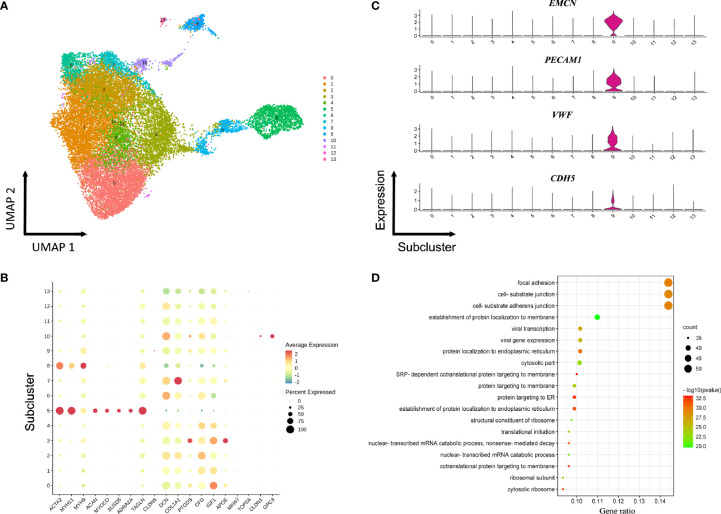
Reclustering of fibroblasts and smooth muscle cells. **(A)** UMAP plot of combined fibroblasts and smooth muscle cells identified *via* non-hierarchical cluster analysis. **(B)** Expression of selected cell-type-specific genes in subclusters. Dot size corresponds to the percentage of cells in a subcluster expressing the gene, and the color is proportional to the gene expression frequency (red represents high expression frequency). **(C)** Violin plots of gene expression demonstrating specifically high expression of *EMCN*, *VWF*, *PECAM1*, and *CDH5* in sC9 fibroblasts. **(D)** GO analysis of the transcriptomic signature in the sC9 fibroblasts subpopulation.

Seven fibroblasts populations and one SMCs population were identified based on unbiased clustering analysis of the raw scRNA-seq data **(** **Figure 1B** **)**. The GO or KEGG functional analysis suggested significant enrichment of extracellular matrix (ECM) organization and cell adhesion **(** **Figures S3**, **S4** **)**. To obtain a clearer identification of cell subpopulations, fibroblasts and SMCs were combined and reclustered into 13 subclusters, including ten subtypes of fibroblasts, two distinct subtypes of SMCs (sC5 and sC8) as well as one subtype expressing endothelial markers (sC9) **(** **Figure 3A** and **Table S4** **)**. The SMCs in the sC5 displayed high levels of cell type-specific markers (*ACTA2*, *TAGLN*, *MYH11*). In contrast, cells in the sC8 co-expressed fibroblasts and smooth muscle markers, which may represent a transition state from SMCs to fibroblasts **(** **Figure 3B** **)**. In comparison with the other subclusters, endothelial signature genes (*EMCN, VWF*, and *PECAM1*) were strongly enriched in a subset of fibroblasts (sC9) **(** **Figure 3C** **)**. GO functional analysis revealed biological processes related to cell junction and focal adhesion, consistent with several functions in the ECs **(** **Figure 3D** **)**. These results perhaps suggested that a cell subpopulation that shared characteristics of ECs and fibroblasts was likely to be involved in the development of ED.

### Differentiation Trajectories Between SMCs and Fibroblasts

**Figure 4:**
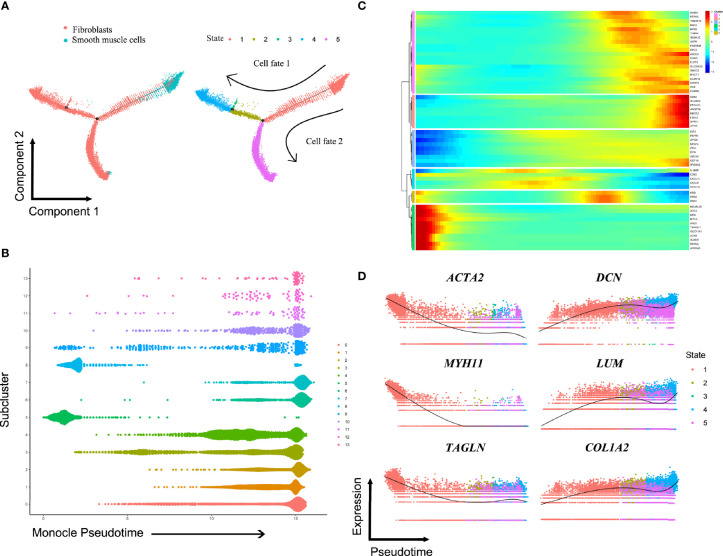
Putative differentiation trajectories from smooth muscle cells to fibroblasts. **(A)** Pseudotime analysis on fibroblasts and smooth muscle cells, arranging them into two major trajectories. **(B)** All cells in subclusters on the pseudotime are color-coded to match the colors in **Figure 3A**. **(C)** Heatmap showing differentially expressed genes among the identified 6 gene clusters. **(D)** Color-coded pseudotime feature plots for selected genes of smooth muscle cells (*ACTA2*, *MYH11*, *TAGLN*) and fibroblasts (*DCN*, *LUM*, *COL1A2*).

Although clustering analysis could reveal heterogeneity among fibroblasts and SMCs in ED tissue samples, it also remained to be determined if they have common differentiation trajectories. The transition trajectory between SMCs and fibroblasts was depicted using Monocle 2 method to identify potential relationships across calculated states. Pseudotime ordering of fibroblasts and SMCs generated 5 states organized into two main branches (**Figure S5**). The predicted pseudotime trajectory began from the right branch and advanced as cells approach the up and bottom left branches, suggesting that SMCs may differentiate into fibroblasts in ED (**Figure 4A**). Consistently, the cells in the sC5 and sC8 (SMCs) were mainly localized in the early stages of pseudotime trajectory while the others (fibroblasts) moved towards the termini (**Figure 4B**). Comparing these two branches, we found that fibroblasts in the sC9 were more likely to go through cell fate 1, whereas the other fibroblasts subclusters did not exhibit preference to fates 1 and 2 (**Figure S6**). Significantly changed genes were assigned to 6 clusters in the trajectory heatmap, demonstrating dynamic gene expression patterns (**Figure 4C**). SMC markers (*ACTA2*, *MYH11*, *TAGLN*) were down-regulated along the pseudotime, peaking in the left of the trajectory. By comparison, marker genes for fibroblasts (*DCN*, *LUM*, *COL1A2*) had an increased expression towards the right of the trajectory (**Figure 4D**).

### Cell Communication and Cell Cycle Analysis

**Figure 5:**
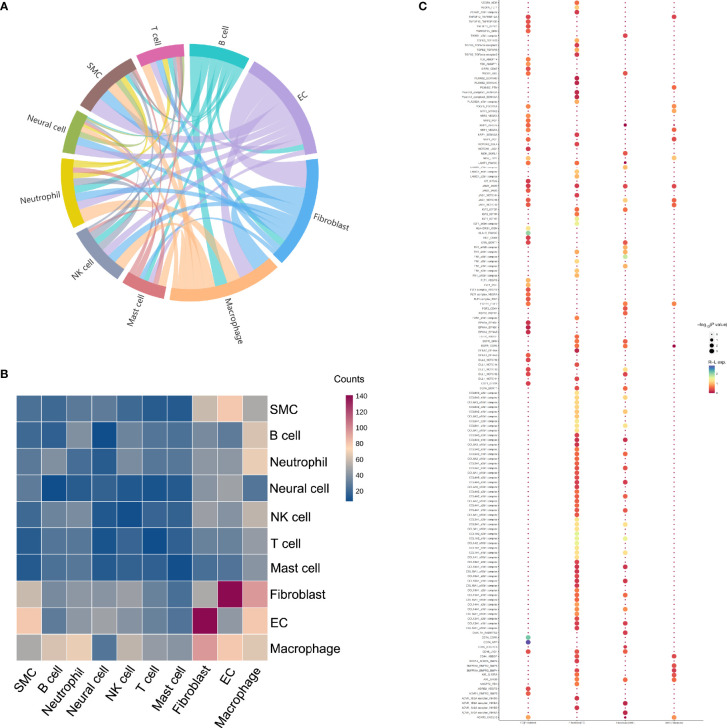
Potential ligand-receptor interactions analyses in different subpopulations. **(A)** The chord diagram shows the quantity of communication among distinct cell types, which are proportional to edge width. **(B)** Heatmap of the number of predicted interactions between cell groups. **(C)** Bubble chart shows the potential ligand-receptor pairs between SMCs and fibroblasts as well as ECs and fibroblasts.

To systematically figure out possible cellular behavior and response to neighboring cells, ligand-receptor pairs were determined between various cell types in ED. The most abundant interactions occurred between fibroblasts and ECs. Moreover, fibroblasts exhibited robust interactions with macrophages. SMCs also showed a medium interaction with macrophages, fibroblasts, and ECs (**Figures 5A, B** **)**. The specific ligand-receptor pairs among SMCs, ECs and fibroblasts were further investigated in detail to analyze their underlying mechanism of cellular communications (**Figure 5C**). For instance, *VEGFA* on fibroblasts was closely bound with *FLT1* and *KDR* on ECs, and many receptors were also involved including *EGFR* and *IGF1R.* The collagen family had a large proportion in interactions predicted for fibroblasts, in which *COL1A2* showed higher expression compared with the other members.

**Figure 6:**
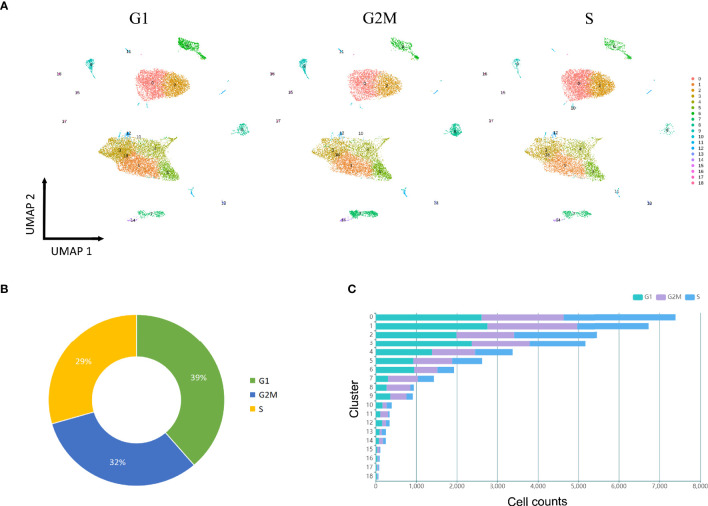
Cell cycle analysis. **(A)** UMAP plot of all clusters at three stages (G1, S, G2M), which are color-coded to match the colors in **Figure 1B**. **(B)** Distribution of cell counts at three stages (G1, S, G2M) in the tissue samples. **(C)** Bar chart shows the cell counts in each cluster at three stages (G1, S, G2M).

To dissect the cell cycle phase in the cavernous tissue, the possible states for each cell cluster were scored using genetic signatures for the G1, S, and G2/M phases. The overall distribution of cell cycle was approximately comparable, yet several clusters had their own patterns (**Figures 6A–C**). The percentage of cells in the G1 phase was increased in the SMCs (C6) and certain fibroblasts (C3, C12, C10). Meanwhile, the G2/M-phase genes were primarily expressed in the T cells (C7), neutrophils (C8), macrophages (C9), and monocytes (C11). Collectively, these findings implicated that severe ED may be associated with abnormal alterations in ECs, SMCs, and fibroblasts, with inflammatory cells engaged in the loss of erectile function as well.

## Discussion

The penile erectile tissues, specifically the cavernous smooth musculature and the smooth muscles of the arteriolar and arterial walls, are essential for the initiation and maintenance of an erection. Any interruption of the hemodynamics and neurophysiology can affect the normal process of penile erection. The cellular composition and molecular profiles of corpus cavernosum have been altered in the development of ED, in which structural changes feature increased vascular resistance, decreased smooth muscle content, impaired endothelium-dependent relaxation, and fibrosis of cavernous tissue (18, 19). Although our understanding of the fundamental mechanisms and pathophysiology have been enhanced in the past decades, many challenges remain to be addressed, especially in severe ED. For example, treatment options in these patients are still limited at present with unsatisfactory clinical outcomes as they are frequently refractory to first-line oral pharmacotherapy. The scRNA-seq platforms can generate notable insights into the cellular diversity through the in-depth profiling of DEGs, emerging as a powerful tool to explore molecular underpinnings in specific physiological and pathological states (7, 20). In recent years, scRNA-seq has broadened our knowledge of various biological processes with immense implications for both basic and clinical research. Therefore, we performed a comprehensive and detailed analysis of human penile samples from patients with severe ED at the single-cell level, providing an opportunity to understand this male sexual dysfunction on a genomic scale.

The vascular endothelium in the penis has a prominent role in modulating vascular tone and blood flow, whose dysfunction has been proposed as a common characterization of ED and also associated with cardiovascular disease (5, 18). As a molecule responsible for initiating physiologic penile erection, NO catalyzed by endothelial nitric oxide synthase in the blood vessel appears essential for vasodilation of penile arteries *via* the NO-sGC-cGMP pathway (21, 22). In our study, pan-endothelial cell markers and other canonical markers are used to identify ECs in the human corpus cavernosum (23–25). The proportion of ECs was largest in the patient with primary vasculogenic ED (P#1). Among the subpopulations of ECs, fibroblasts-like and arterial ECs have demonstrated distinct transcriptional profiles compared with the others. The primary source of penile blood consists of three paired arteries (cavernous, dorsal, bulbourethral), which supply the trabecular erectile tissue and the sinusoids. We have found that a unique cell subtype of ECs has co-expressed fibroblasts markers. Similarly, the clustering analysis of fibroblasts and SMCs implied the existence of an endothelial subcluster. Their cellular differentiation trajectories were also analyzed, indicating that ECs were possible to transform into fibroblasts (**Figure S7** **)**. Taken together, these findings suggested potential crosstalk between ECs and fibroblasts in the pathogenesis of ED. In the endothelial clusters (C0, C2, C13), focal adhesion and protein localization are mainly enriched functions, especially compact cellular junctions in arterioles; in contrast, their physiological roles in angiogenesis and cell migration have not been apparent in severe ED. The heterogeneous phenotypes of ECs have been investigated in multiple tissues and diseases, showing that vascular ECs have transcriptome similarity across tissues but vary substantially in different pathological states (26–28). Many genes that are specifically expressed in ECs subpopulations other than arterial ECs can regulate ECM organization and the formation of caveolae such as *MMRN1* and *CAVIN2* (29, 30). The other genes warrant further study to enhance our understanding of ECs in the settings of ED.

Along with the ECs that form the interior surface of blood vessels (tunica intima), SMCs and fibroblasts are also primary components of the vascular wall, which constitute the middle (tunica media) and outer section (tunica externa), respectively. Our experiments revealed the mean proportion of fibroblasts was more than 50% in the corpus cavernosum of ED. In particular, the patient who suffered from pelvic trauma (P#3) had the largest number of fibroblasts, consistent with their functions in tissue homeostasis and wound healing (31). These results have suggested that ED caused by injury or trauma could mostly be attributed to cavernous fibrosis while endothelial dysfunction has been relatively crucial in primary vasculogenic ED. Penile fibrosis has been thought to be a diffuse process related to conventional risk factors and etiological factors for ED (32). As a consequence, smooth muscle contraction and relaxation would be damaged, making the penis incapable of becoming completely rigid and impairing patients’ quality of life to a certain degree. The fibrotic process of the corporal spongy tissue occurs based on intricate molecular pathways and cellular interactions, yet little research has been directed to this benign disease until now. The scRNA-seq data in our analysis uncovered the heterogeneity of fibroblasts and SMCs as well as predicted trajectory of differentiation, suggesting that corpora cavernosa fibrosis is possibly responsible for the development of ED. The aberration of many developmental pathways has been highlighted in the KEGG analysis, including PI3K-Akt, MAPK, and Ras signaling pathways. Multiple regulatory mechanisms have been participated in the differentiation of SMCs, including TGF-β signaling, Notch signaling, and epigenetic regulation (33). The transition of healthy SMCs into myofibroblasts can be induced in Peyronie’s disease, which may share a similar mechanism with ED (34). Co-expression of *PECAM1*, *VWF*, and *EMCN* in one subpopulation suggests differentiation into ECs-like fibroblasts from part of SMCs *via* a distinct cell fate. The scRNA-seq analysis of vascular malformations revealed that a small fraction of SMCs had the capacity to be transformed into ECs and fibroblasts (35). Consequently, our results suggest that extensive penile fibrosis that develops in the human corpus cavernosum can contribute to severe ED.

Complex cell-cell communication networks are of great significance for basic cellular activities and coordination of cell actions. Abnormal cell signaling may cause pathological disease progressions such as endothelial dysfunction, diabetes, and cancer (36–38). Furthermore, extracellular vesicles deliver many functional molecules for intercellular communication in several male diseases (39). Gene expression measurements of our scRNA-seq datasets have shown strong interactions among macrophages, fibroblasts, SMCs, and ECs, emphasizing the role of these cells in the cavernous tissue in ED. The fibroblasts-related ligand-receptor pairs mainly center on collagenization of the smooth muscle and endothelium. Of note, the ligand-receptor expression of CD74-related pairs was increased in ECs, B cells, and macrophages, such as the interaction with secreted amyloid precursor protein. Previous studies have demonstrated that various regulatory and trophic factors can influence the local penile tissue environment, including vascular endothelial growth factor, tumor growth factor, and insulin-like growth factor (40–42). Le Hiress et al. reported that the CD74 signaling system was critical for a phenotypic swift to proinflammatory ECs in patients with pulmonary arterial hypertension (43). As pulmonary hypertension and ED can both be treated with inhibitors of phosphodiesterase 5, CD74 may be another highly potent target for ED. More evidence is needed to tentatively confirm this hypothesis, shedding further light on the molecular biology and signal transduction of penile erection.

Several limitations of our study should be outlined to optimize and expand scRNA-seq datasets for ED. First, a relatively small number of patients with severe ED were recruited owing to the difficulties of obtaining human penile tissues. In addition, the single-cell suspension of certain samples was excluded as they did not meet the requirements of quality control procedures. Second, lack of healthy controls means that the comparison between ED and normal tissues was not feasible in our study. Third, another limitation mainly lies in the validation of our findings. Generally speaking, many novel computational approaches are still in their infancy and the biological interpretations of findings are heavily biased by the researcher’s familiarity with studied topic. With the recent maturation in scRNA-seq and the development of powerful analysis tools such as Human Cell Atlas, our understanding of ED-associated cellular functions will be increasingly improved in the future.

## Conclusions

In conclusion, we performed a scRNA-seq analysis of penile cavernous tissue from patients with severe ED, providing detailed expression profiles of cellular subsets. Our data suggest that penile fibrosis and inflammation have been noteworthy characteristics in the late stage of ED, which may offer deep insight into the erectile process and serve as an essential resource for targeted therapy for this common male sexual dysfunction.

## Data Availability Statement

All data generated or analyzed during this study are included in this published article and its **Supplementary Material**, which are available on reasonable request. The raw sequence data generated in this study have been deposited in the National Omics Data Encyclopedia (NODE) database (www.biosino.org/node) under accession codes OEP002391, OEP002948, and OEP003055.

## Ethics Statement

This study involving human participants was in accordance with the ethical standards of the institutional and national research committee and with the 1964 Helsinki Declaration and its later amendments or comparable ethical standard. The protocol was approved by the Institutional Review Board of Peking University First Hospital.

## Author Contributions

Conception and design: DF and R-LG. Administrative support: Z-CZ, Z-CX, and X-SL. Collection and assembly of data: W-PS, Y-YG, J-CP, X-QY, W-DS, Y-MY, and JP. Data analysis and interpretation: DF and X-HT. Manuscript writing: DF and X-HT. Final approval of manuscript: All authors.

## Funding

Supported by the National Natural Science Foundation of China (grant no. 82171610, 81971379, 82001534) and Beijing Natural Science Foundation (grant no. 7202204).

## Conflict of Interest

The authors declare that the research was conducted in the absence of any commercial or financial relationships that could be construed as a potential conflict of interest.

## Publisher’s Note

All claims expressed in this article are solely those of the authors and do not necessarily represent those of their affiliated organizations, or those of the publisher, the editors and the reviewers. Any product that may be evaluated in this article, or claim that may be made by its manufacturer, is not guaranteed or endorsed by the publisher.

## Abbreviations

cGMP, cyclic guanosine monophosphate; DEGs, differentially expressed genes; ECM, extracellular matrix; ED, erectile dysfunction; ECs, Endothelial cells; GO, Gene Ontology; KEGG, Kyoto Encyclopedia of Genes and Genomes; NO, nitric oxide; PBS, phosphate-buffered saline; sGC, soluble guanylate cyclase; scRNA-seq, single-cell RNA sequencing; SMCs, smooth muscle cells; UMAP, uniform manifold approximation and projection; UMI, unique molecular identifiers.
