# Supplementary material for: Single-Cell RNA Sequencing of Human Corpus Cavernosum Reveals Cellular Heterogeneity Landscapes in Erectile Dysfunction
Source: Front Endocrinol (Lausanne). 2022 Apr 20;13:874915. doi: 10.3389/fendo.2022.874915 (PMC9066803; doi:10.3389/fendo.2022.874915)
Supplement: Supplementary file 1 [file Presentation_1.pdf]

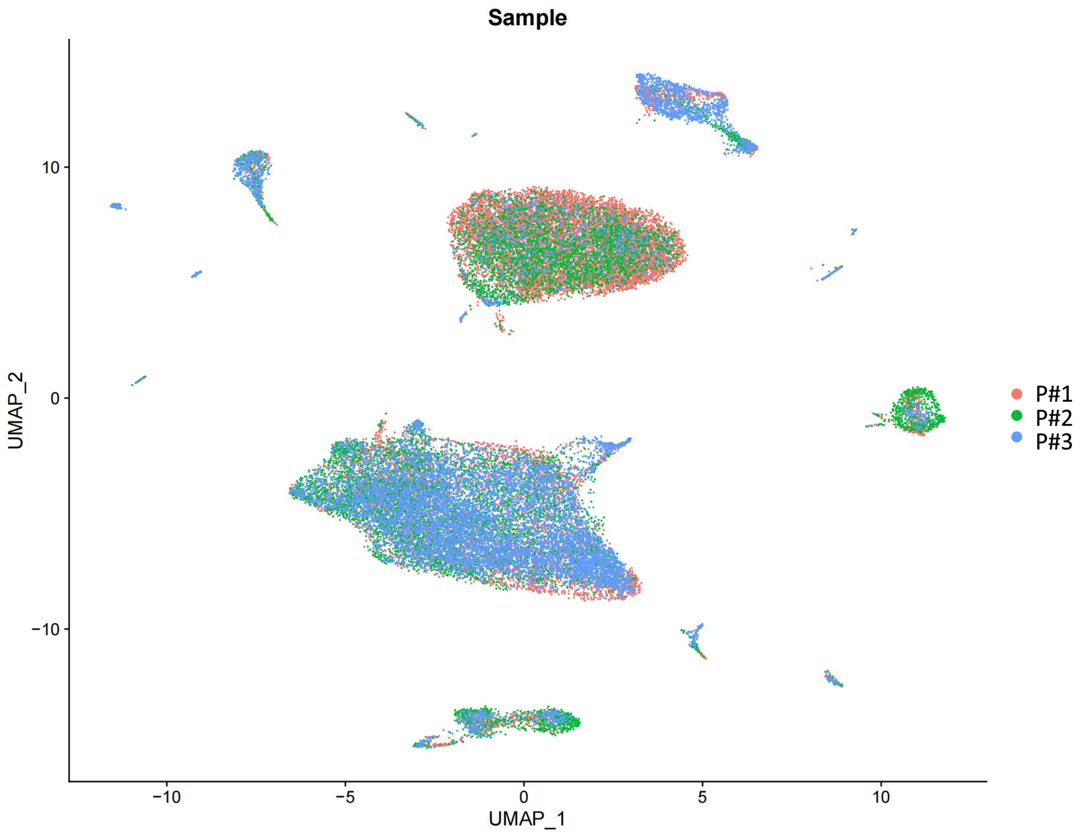

Figure S1. UMAP plot of three samples were colored as indicated in the legend.

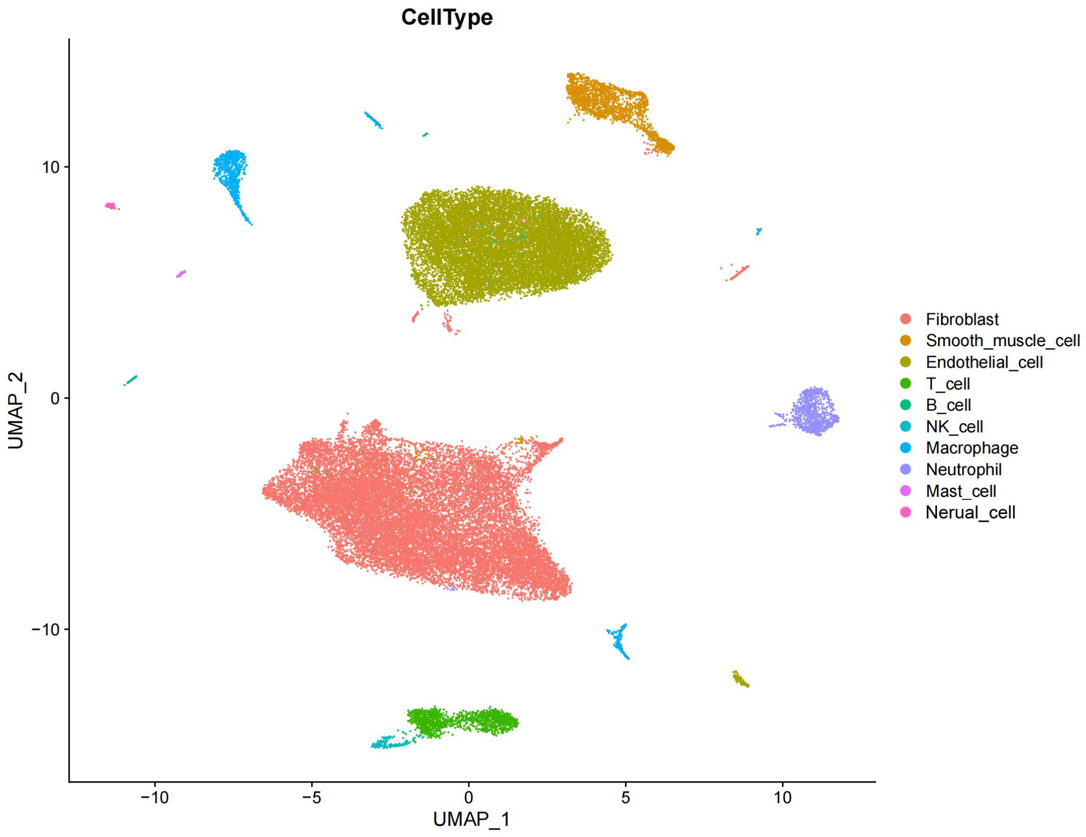

Figure S2. UMAP plot of cells from the three samples profiled in this study, with each cell colored coded to indicate the associated cell types.

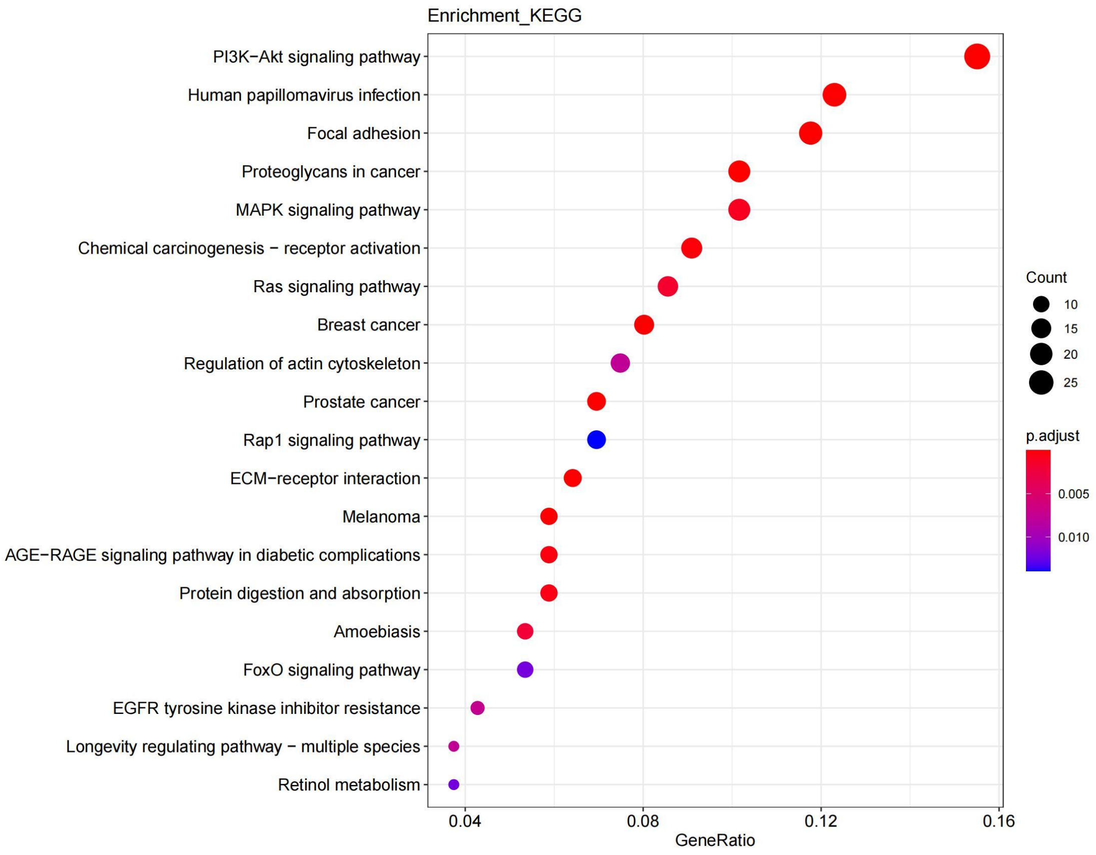

Figure S3. KEGG analysis of fibroblasts, type I (Fib I)

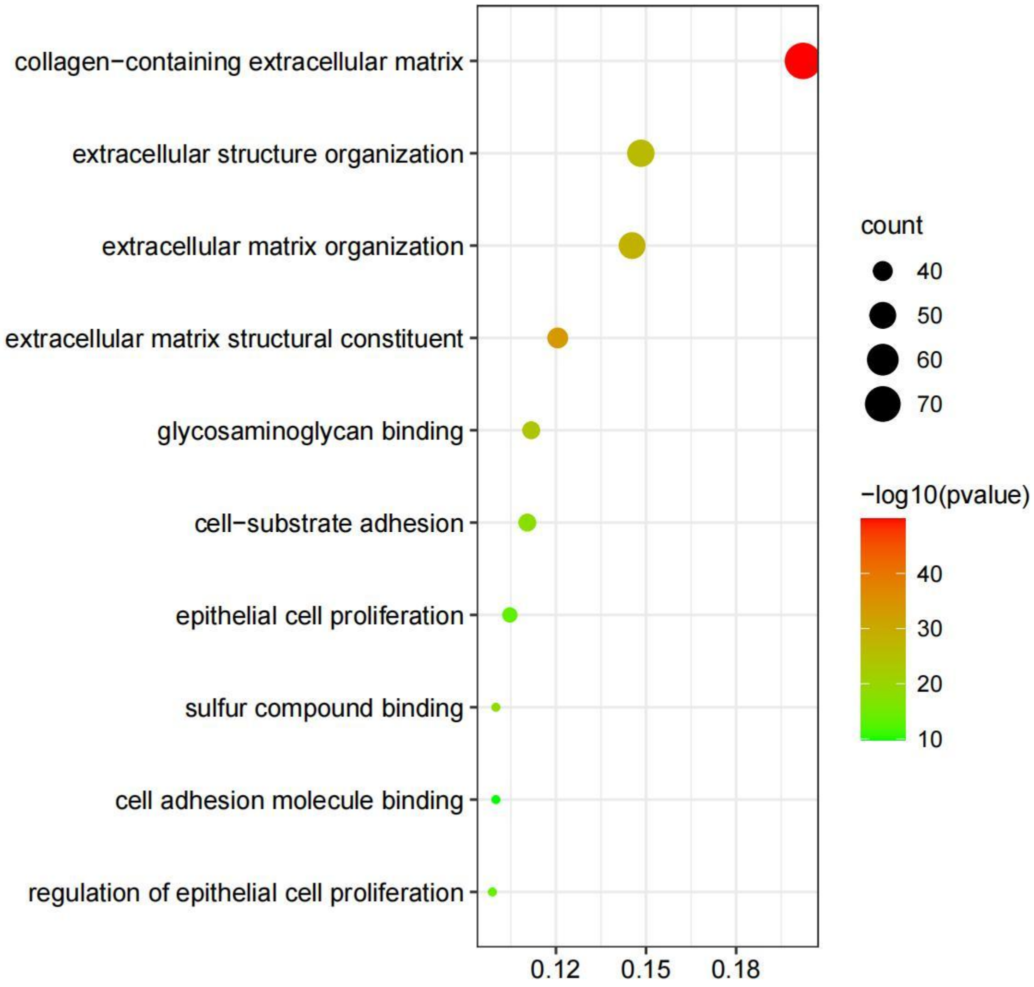

Figure S4. GO analysis of fibroblasts, type I (Fib I)

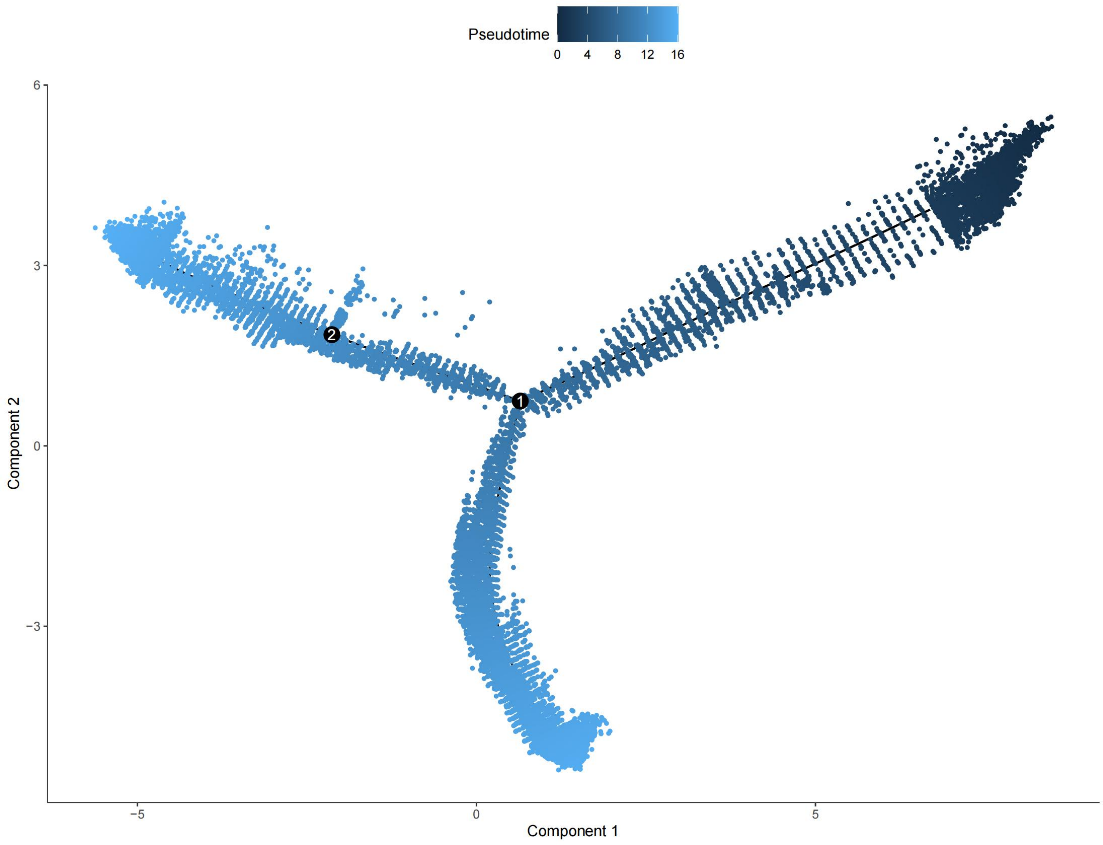

Figure S5. Cells are colored based on pseudotime inferred from cell trajectory, with pseudotime beginning in the branch on the right and advancing as cells approach two left branches.

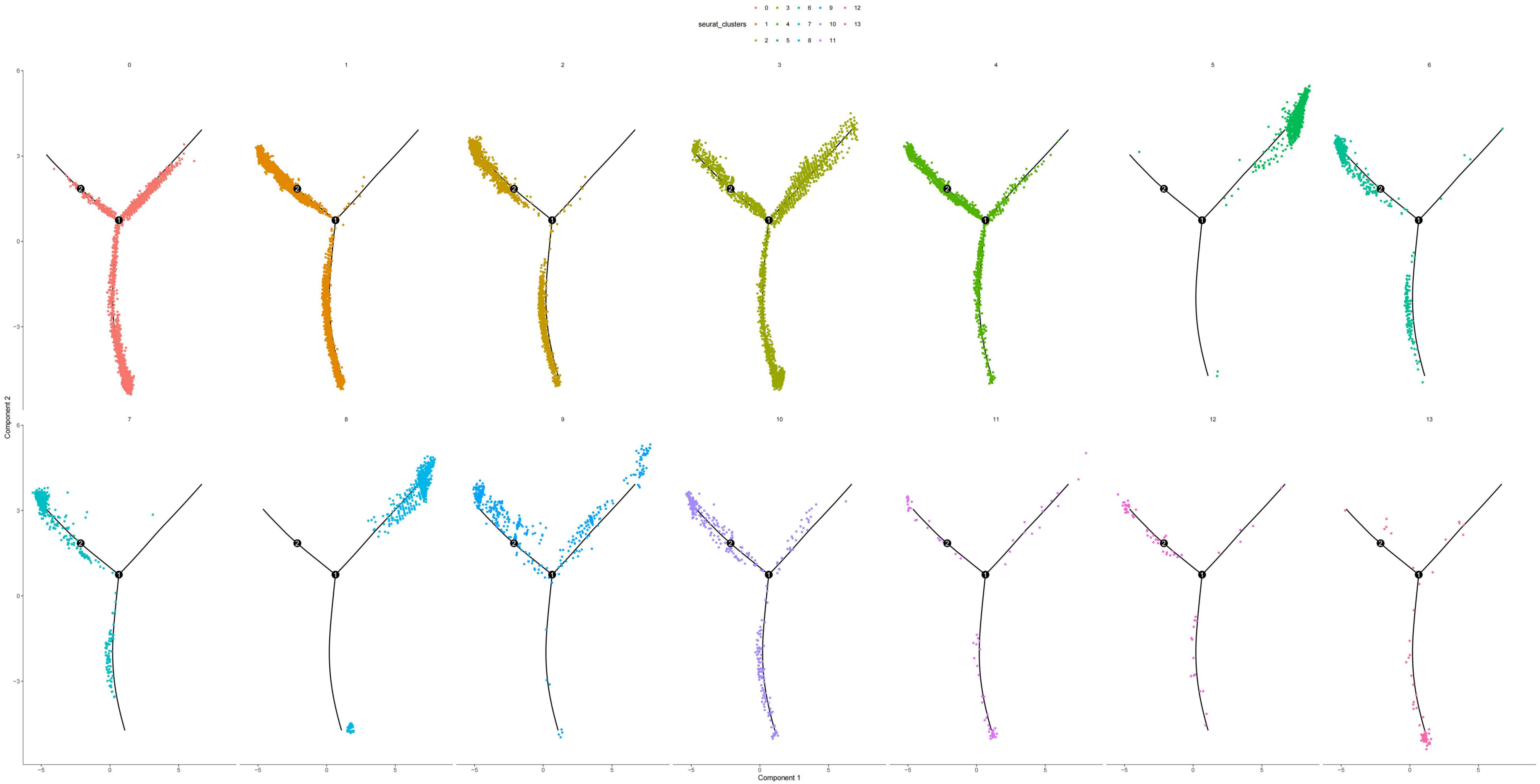

Figure S6. Cells from distinct subclusters shows their relative abundance across the pseudotime trajectory

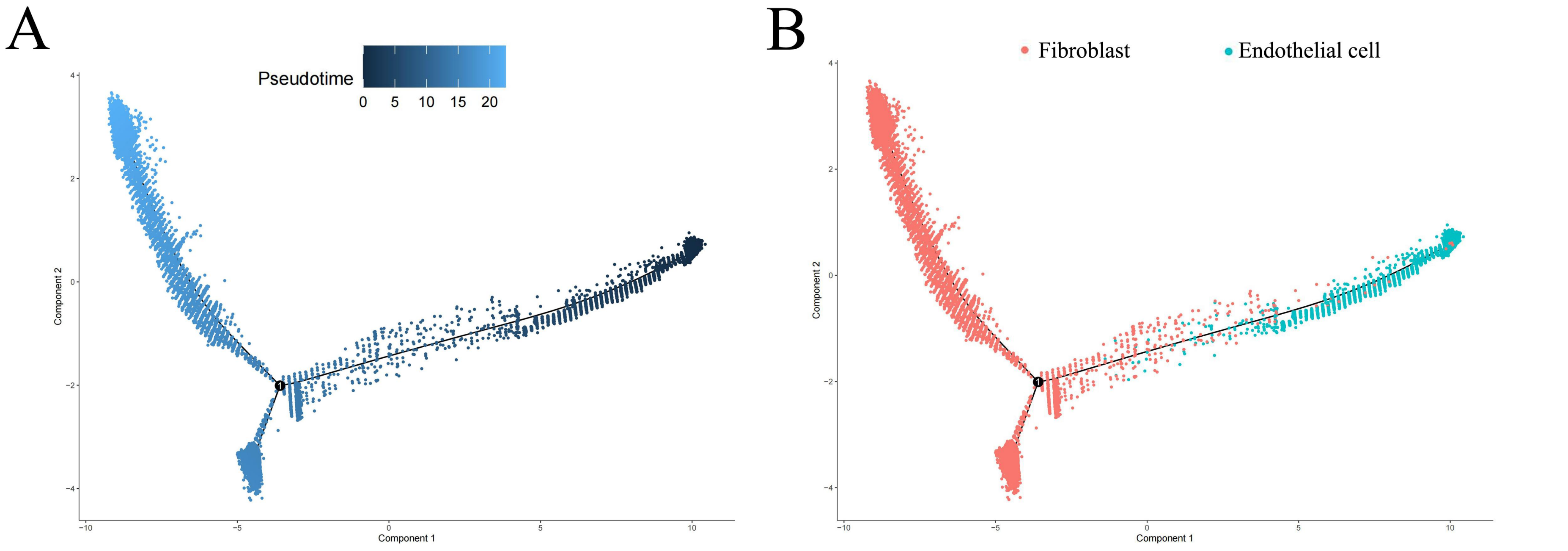

Figure S7 Predicated differentiation trajectories from endothelial cells into fibroblasts

The discriminative dimensionality reduction with trees algorithm was performed between endothelial cells and fibroblasts. (A) Cells are colored based on pseudotime inferred from cell trajectory, which corresponded to Figure S5. (B) The cell types were colored as Figure 4A.
